# Discrepancies in regulations in post-marketing safety surveillance of drug-device combination products in the EU and US: a review

**DOI:** 10.3389/fdsfr.2025.1609455

**Published:** 2025-11-26

**Authors:** Joan D’souza, Mukesh Nandave, Ruth Blöchlinger

**Affiliations:** 1 JD Pharma Services, Zurich, Switzerland; 2 Department of Pharmacology, Delhi Pharmaceutical Sciences and Research University (DPSRU), New Delhi, India; 3 Recordati Pharma, Milan, Italy

**Keywords:** drug-device combination products, postmarketing surveillance, drug safety, drug delivery systems, regulatory harmonization, pharmacovigilance

## Abstract

This article captures the differences in regulations in post-marketing safety surveillance in drug device combinations in the US and the EU. Drug-device combinations have improved the landscape of health care through both drug effectiveness and personalized medicine. Regulatory frameworks in the U.S. and E.U. ensure these products are safe and effective. In the E.U., combination products are officially regulated according to their primary mode of action (PMOA) as per EU MDR 2017/745 and MPD 2001/83/E.C., executed by the European Medicines Agency (EMA) and National Competent Authorities (NCAs). Products are classified as integral, co-packaged, or cross-labeled, with extensive pre-marketing assessment and post-marketing surveillance. In the United States, the combination products are regulated per 21 CFR 3.2(e) by the Office of Combination Products (OCP) and lead centers (CDER, CDRH, CBER), which ensure adequate compliance. Post-marketing surveillance, risk management, and safety signal identification, are key features of the FDA’s Adverse Event Reporting System (FAERS) and Risk Evaluation and Mitigation Strategies (REMS). While the E.U. and U.S. have robust regulatory frameworks for drug-device combination products, they differ in their approaches, leading to challenges in global harmonization. In the EU, no single regulatory authority oversees the entire lifecycle of combination products. The MDR focuses on device performance and risk management while the EMA focuses on medicinal product efficacy and safety. The drug safety reporting system EudraVigilance focuses on the medicinal component, with EUDRAMED. In the U.S., the FDA oversees these products, with the Center for Drug Evaluation and Research (CDER) or the Center for Devices and Radiological Health (CDRH) determining the regulatory path whether the product acts primarily as a drug or a device. Misalignment between the EU and US frameworks creates issues for global harmonization, affecting access, manufacturing, and patient safety. International cooperation and the implementation of standardized guidelines are crucial for advancing global healthcare and ensuring the efficacy and safety of drug-device combination products. This article aims to discuss discrepancies in regulation between the US and EU and discuss the feasibility of the current problems.

## 1 Introduction

### 1.1 Background on drug-device combination products

Drug-device combination products are defined as devices that are enriched with drugs or biological products to offer synergistic clinical management. These products are the outcomes of the research and development that reflect the evolution and integration of modern-day pharmaceuticals and medical devices to attend to complex healthcare needs of patients. Drug-device combination products have resulted in significant advancements in medical technologies that have improved and evolved diagnostic and therapeutic outcomes, offering enhanced patient care with innovative treatments (Bayarri, 2015). From revolutionary drug-eluting coronary stents (Lee and de la Torre Hernandez, 2018) to pre-filled syringes and the use of inhalers, drug-device combinations have enhanced efficacy (Lavorini et al., 2017), targeted delivery, eased administration, added convenience, and enabled compliance. Our healthcare has come one step closer to the development and execution of the longstanding vision of personalized medicine.

**FIGURE 1:**
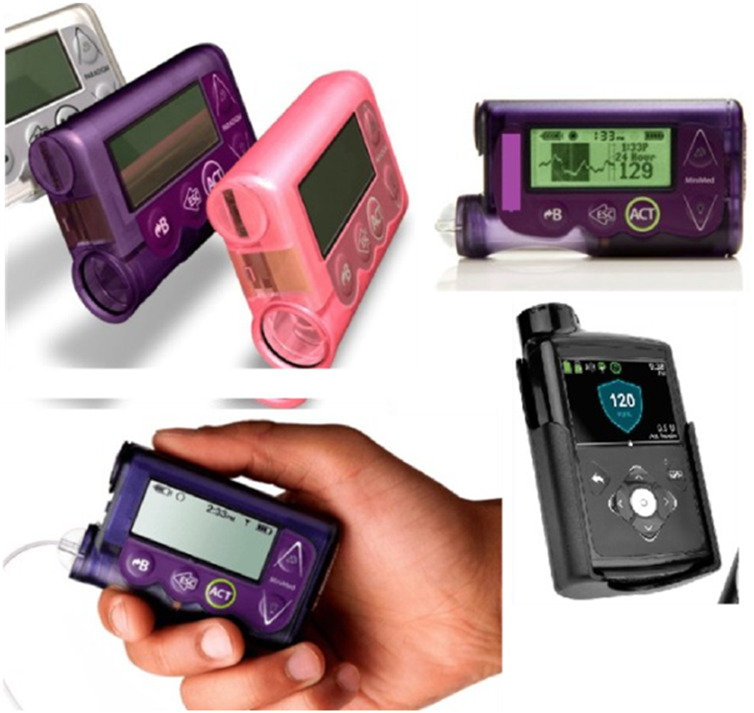
Examples of Mini-med insulin pumps.

**FIGURE 2:**
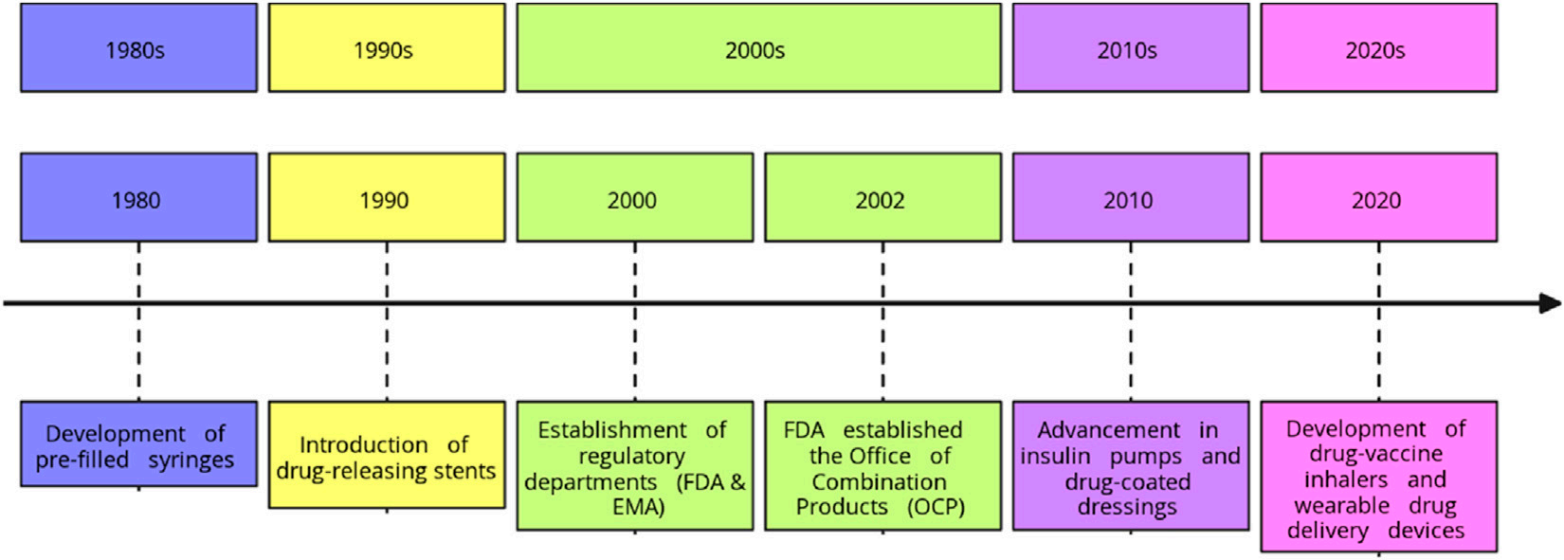
Timeline of the events related to drug-device combination products.

The development of drug-device combinations began in the 1980s in the form of pre-filled syringes. In the 1990s, drug-releasing stents significantly improved the management of cardiovascular diseases by reducing the risks of restenosis (Htay and Liu, 2005). In the early 2000s, this development in the sector of integrative medicine resulted in the establishment of the regulatory departments in the U.S. Food and Drug Administration (FDA) and European Medicines Agency (EMA) that implemented specific guidelines to oversee the development of combination products. The FDA established the Office of Combination Products (OCP) in 2002 to regulate, implement and check the compliance of the products with the guidelines (Reis et al., 2022). The regulations have influenced the growth and sophistication of combination products as we have seen in the form of insulin pumps to manage diabetes (Figure 1), drug-coated dressings for improved wound healing, and more recently drug-vaccine inhalers and wearable drug delivery devices. The timeline of the important events is summarized below (Figure 2).

### 1.2 Importance of post-marketing safety surveillance

Despite the praiseworthy advancement of this sector, it must be considered that the integration of different mechanisms in a single device is more prone to errors that are essential to be monitored and kept under surveillance. The regulatory bodies must identify and report the risks of combination products that may not have been evident during pre-market testing.

Post-marketing safety surveillance is essential to detect and report the long-term adverse effects associated with the use of combination products that are majorly missed in short-term pre-market studies. Since clinical trials often include small sample sizes, post-market surveillance helps in the identification of rare and infrequent events that were undetected in clinical trials. Furthermore, clinical trials are usually performed in controlled clinical environments and, therefore, might not reflect the same in broader, diverse and varied real-world conditions (Alomar et al., 2020; Raj et al., 2019). The reporting and troubleshooting of resulting observed discrepancies, in addition to device malfunction or user errors, are the responsibilities of the regulatory bodies that oversee the post-safety surveillance (Alomar et al., 2020). The importance of post-marketing safety surveillance can be understood through numerous recent case studies. For instance, the clinical trials of Paclitaxel-eluting stents reported a promising reduced restenosis rate compared to the bare-metal stents; however, post-market surveillance revealed unexpectedly increased rates of late thrombosis and associated mortalities (Mathlouthi et al., 2021). A similar case happened with Medtronic’s Mini-med insulin pumps, which were designed to deliver controlled dosages of insulin to manage diabetes. Still, the flaws identified in the retainer ring of the pumps resulted in delivery of improper dosage of insulin. This posed a significant risk of hyper- or hypoglycemia; therefore, the product was recalled (Klonoff and Han, 2019).

Another relevant case involves the Advair Diskus, an inhaler combining a corticosteroid (fluticasone) and a long-acting beta-agonist (LABA), salmeterol, to manage asthma and chronic obstructive pulmonary disease (COPD). Although approved for its dual action in reducing inflammation and relaxing airway muscles, post-marketing data revealed that LABAs like salmeterol might increase the risk of asthma-related deaths. In response, the FDA mandated a boxed warning on all LABA products and required manufacturers to implement additional safety measures, such as ensuring that LABAs are only prescribed alongside corticosteroids, to mitigate these risks (Miller-Larsson and Selroos, 2006). This example highlights the critical role of regulatory bodies in enforcing strict post-marketing safety surveillance and guidelines to protect patient safety. Table 1 details the importance of each aspect of post-marketing safety surveillance in minimizing risks associated with combination products.

**TABLE 1:** Various Aspects of Post-marketing surveillance and limitations.

Aspect	Description
Prone to errors	Integration of different mechanisms in a single device increases the likelihood of errors, necessitating pre-marketing safety surveillance
Role of regulatory bodies	Responsible for identifying and reporting risks of combination products not evident during pre-market testing
Essential surveillance	Both post-marketing safety surveillance (FDA) or post-market surveillance (EU) detects and reports long-term adverse effects missed in short-term pre-market studies
Clinical trials limitations	Small sample sizes in clinical trials may miss rare and infrequent events; controlled environments may not reflect real-world conditions
Regulatory responsibilities	Includes reporting and troubleshooting observed discrepancies, device malfunctions, and user errors

### 1.3 Purpose of the review

Considering such cases and the growing industry of combination products, it has become essential to understand the importance of the regulations and transparency of regulatory bodies in ensuring the efficacy and safety of these products. For that purpose, the regulatory bodies must work collaboratively to employ rigorous pre-market assessment and robust post-marketing surveillance tactics not only to monitor but also to attend to adverse events in the view of the protection of public health. In this review, we aim to introduce the combination products subject to the regulatory framework followed by an overview of the major regulatory bodies like the FDA and EMA, their pharmacovigilance system, post-surveillance risk management, critical discrepancies between them and finally, the challenges faced with the regulation.

### 1.4 Overview of the regulatory frameworks in the E.U. and US

**FIGURE 3:**
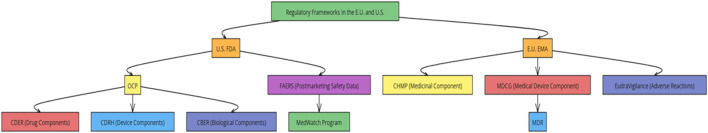
Summary of the regulatory frameworks in EU and US

With the global rise in the utilization and applications of drug-device combination products, regulatory bodies have been working towards the efficacy, safety, marketing and risk management of the drug-device combination products. These regulatory bodies include the globally recognized U.S. FDA and E.U.’s EMA. Others of national importance include the Pharmaceuticals and Medical Devices Agency (PMDA) in Japan, the Therapeutic Products Directorate (TPD), the Medical Devices Directorate (MDD) and the Marketed Health Products Directorate (MHPD) in Canada, and the Therapeutic Goods Administration (TGA) of Australia. Additionally, the International Council of Harmonization of Technical Requirements for Pharmaceuticals for Human Use (ICH) and World Health Organization (WHO) play instrumental roles in ensuring quality, safety, compliance, and risk management at global levels, especially in developing nations that lack their standardized regulator bodies (Teixeira et al., 2020). Among these, the United States’s FDA and the European Union’s EMA are well-established regulatory bodies that, through their meticulous policies and implementations, have been successful in achieving global trust and recognition. In 2002, the U.S. FDA established OCP, which, through its lead centers, coordinates the required mode of action to oversee and review the particulars of drug-device combination products. The Center for Drug Evaluation and Research (CEDR) reviews the drug components of the combination products. In contrast, the device component is checked by the Center for Devices and Radiological Health (CDRH). The Center for the Biologics Evaluation and Research (CBER) oversees, manages and regulates the particulars of the associated biological components. Once the product is launched into the market, the FDA’s Adverse Events Reporting System (FAERS) is responsible for the collection of post-marketing safety data that, in association with the MedWatch program, is being reported to the respective centers of the FDA (Manresa and Meyers, 2005). Similarly, the European Union member states, apart from their own National Competent Authorities (NCAs) (Europ ean Medicines Agency, 2025), are collectively in compliance with a standard regulatory body, the European Medicine Agency (EMA). The EMA has a few major regulatory centers: the Committee for Medicinal Products for Human Use (CHMP), which reviews the medicinal component of the combination product; the Medical Device Coordination Group (MDCG), which functions under Medical Devices Regulation (MDR) to oversee the medical device part, and finally the EudraVigilance system that has been established to manage and analyze the information on the suspected adverse reactions to the combinational devices (European Medicines Agency, 2025). A summary of the regulatory frameworks in EU and US has been shown in Figure 3.

## 2 Regulatory frameworks for drug-device combination products

### 2.1 Definition and classification of combination products

#### 2.1.1 E.U. perspective

In the European Union, the combination product is defined and categorized based on its primary mode of action (PMOA). The EU MDR defines combination products as the integration between a medical device, a medicinal compound, and a biological element. The EMA oversees the regulation of medicinal devices and works in collaboration with NCAs for evaluation, review, and pre-/post-marketing surveillance of the combination product. The EU MDR 2017/745 and MPD 2001/83/E.C. provide the necessary regulatory framework for the combination products in the E.U. (Reis et al., 2022). Based on their guidelines, the combination products can be classified as medicinal products with an integral medical device where products like drug-pre-filled syringes or inhalers form a single integrated product (De Man et al., 2021). However, in the case of medical devices with an adjuvant medicinal substance like heparin-coated blood oxygenators or antimicrobial-coated catheters, the medicinal part is ancillary to the primary function of the medical device. Co-packaged and cross-labelled products form the third classification, where the device and medicinal part are physically separated in the same package but are instructed to be used together; for example, the drug and its delivery device are sold together but combined at the time of its application (De Man et al., 2021).

#### 2.1.2 U.S. perspective

In the United States, a drug-device combination product is defined under 21 CFR 30(e) as any combination of drug, device, and biological product. The classification of the combination products also remains the same as that of the E.U. counterpart, including the single-entity combination product, where the medicinal component is fused with the medical device intended to be used together. Then there are co-packaged combination products that include the kits where the medicinal part and the delivery device are packed together but combined at the time of use. Finally, cross-labelled combination products are sold separately but are supposed to be boughttogether for the application. In the U.S., the FDA’s OCP with their respective lead centers (CDER, CDRH, and CBER) decide, review the primary mode of action and evaluate the pre-/post marketing surveillance of the combination product (Maude Manual, 2025).

While the focus of both the E.U. and FDA is to ensure the efficacy and safety of the drug-device combination products, their approach to doing so differs in terms of regulation and policies. In the EU there is no centralized authority for combination products. Based on the rules set by the EMA and the MDR in association with notified bodies and NCAs, these agencies have been competent enough to oversee the framework and guidelines associated with the success of combination products. Conversely, the U.S. FDA has centralized oversight of regulatory aspects of the combination product under the FDA. The OCP with its lead centers specialized to ensure the proper review, plan of action, and monitoring of the products, both pre-and post-marketing stages (Büyüksagis and Werro, 2021). Post-marketing safety surveillance forms a critical component of the regulatory framework to ensure the long-term efficacy and safety of the combinational product. Surveillance is a multi-process system that involves data collection, analysis, and management of the risks associated with the adverse events reported in a combination product.

An overall summary of the differences between two perspectives are shown below in Table 2.

**TABLE 2:** Differences between US and EU perspective.

Parameter	EU perspective	US perspective
Definition and classification	Defined based on primary mode of action (PMOA)	Defined under 21 CFR 3.2(e) as any combination of drug, device, and biological product
Regulatory framework	EU MDR 2017/745 and MPD 2001/83/E.C.	FDA’s Office of Combination Products (OCP) with respective lead centers (CDER, CDRH, CBER)
Oversight body	European Medicines Agency (EMA) in collaboration with National Competent Authorities (NCAs)	U.S. Food and Drug Administration (FDA)
Primary regulatory documents	EU MDR and MPD.	FDA regulations under 21 CFR.
Product classification	- Integral products (e.g., drug-pre- filled syringes, inhalers)	- Single-entity combination products (medicinal component fused with medical device)
	- Medical devices with ancillary medicinal substances (e.g., heparin- coated blood oxygenators, antimicrobial-coated catheters)	- Co-packaged combination products (kits with medicinal part and delivery device packed together)
	- Co-packaged and cross-labelled products (device and medicinal part separated but used together)	- Cross-labelled combination products (sold separately but used together)
Evaluation and surveillance	EMA and NCAs evaluate, review, and conduct pre-/post-marketing surveillance	OCP and lead centers (CDER, CDRH, CBER) decide, review primary mode of action, and conduct pre-/post-marketing surveillance
Post-marketing Safety surveillance	Multi-process system involving data collection, analysis, and risk management of adverse events associated with combination products	Ensures long-term efficacy and safety through data collection, analysis, and risk management of adverse events associated with products

## 3 Post-marketing safety surveillance in the E.U.

### 3.1 Pharmacovigilance systems

The pharmacovigilance system oversees the activities related to the identification, analysis, understanding, and prevention of adverse effects of a medicine or medicinal device. Every drug regulatory body has a functional surveillance system and pharmacovigilance system of EU EMA that looks after and monitors the drug-led combination products is EudraVigilance.

#### 3.1.1 EudraVigilance

EudraVigilance or European Union Drug Regulating Authorities Pharmacovigilance is a centralized pharmacovigilance-based network and management system authorised by the European Economic Area (EEA). It collects and analyses the data on adverse reactions (ADRs) for medicines and drug-led medical devices. EudraVigilance monitors and keeps reports on both clinical and post-marketing safety surveillance Data on the adverse drug reactions is collected from mandatory reports by healthcare professionals and market authorization holders or even reports from patients. This data obtained from various sources is analysed to set safety signal thresholds. Once signals are validated, they are investigated further to determine risks, which can result in updates to risk management plans (RMPs) or regulatory actions. All the data from the EudraVigilance is shared with NCAs and other stakeholders to spread the awareness warranting assessment and management further to ensure drug safety (Pinheiro et al., 2016).

EUDAMED, is a centralized database currently under phased implementation, primarily designed for medical devices under the Medical Device Regulation (MDR) (EU 2017/745) and *In Vitro* Diagnostic Regulation (IVDR) (EU 2017/746). It plays a supporting role in the oversight of drug-led combination products. While the European Medicines Agency (EMA) governs the medicinal product component, EUDAMED manages key aspects related to the device component in these products. EUDAMED complements EudraVigilance by managing device-related incidents, providing a broader view of the product’s safety. The EU system is still evolving, with EUDAMED aiming to unify and harmonize device reporting, though itremains fragmented. These dual systems (EUDAMED for devices, EudraVigilance for drugs) that operate independently, creating potential redundancies.

#### 3.1.2 Role of the EMA and national competent authorities

EMA plays an instrumental role in the regulation of the policies and procedures associated with the approval of the combination product. EMA is responsible for coordination activities that include overseeing the EudraVigilance system to provide the pharmacovigilance platform across the E.U. The EMA analyses the data obtained from the surveillance to offer essential recommendations on risk management measures. Additionally, EMA provides guidelines and good pharmacovigilance practices (GVPs) (European Medicines Agency, 2024)for safe reporting. The regional authorities of the European Union, also known as National Competent Authorities (NCAs), also support EMA in monitoring the safety associated with medicines and medical devices via continuous data sharing with EudraVigilance (Postigo et al., 2018). Furthermore, to ensure the compliance of the medicine and medical device marketing authorization holders, NCAs conduct pharmacovigilance inspections. Also, NCAs perform the critical function of broadcasting safety information to the public, especially the ones recommended by the EMA post-assessment of the collected safety data (Potts et al., 2020).

### 3.2 Adverse event reporting requirements

It is the responsibility of the marketing authorization holders, patients, and healthcare professionals at all levels to report both adverse events and adverse drug reactions. This data works like a feedback mechanism that is essential to ensure proper monitoring of combination product safety (Mammì et al., 2013). It is recommended that the marketing authorization holders must report severe adverse drug reactions within the first fortnight of their awareness; meanwhile, for non-serious adverse drug reactions, the reporting time must be under 3 months. The reporting has a particular format that must be filled with detailed information about the patients, the applied product, the suspected reaction, the nature of the event (seriousness level), and the associated outcomes. Once the report has been drafted, the data must be shared with EudraVigilance via electronic media for further risk assessment, followed by public awareness (Potts et al., 2020).

### 3.3 Risk management plans (RMPs)

After reporting the suspected adverse events, the next step is to assess the risk of the event, followed by the development of risk management plans (RMPs). RMPs include a meticulous process of identification, characterization, prevention and minimization of the risks associated with medical products. Marketing authorization holders majorly develop these plans to mitigate the related risks associated with their products. RMPs include the provision of the safety profile of the medicinal product that involves its known or associated potential risks. Also, healthcare professionals must have a guide or outline to monitor, evaluate, and report on the product’s safety. Furthermore, in order to reduce the suspected adverse effects, product information like proper labelling, education materials, and restricted distribution must be ensured (Butler et al., 2021).

### 3.4 Periodic safety update reports (PSURs)

In addition to reporting adverse events, marketing authorization holders are required to submit comprehensive reports of the medicinal product safety status at regular intervals in the form of periodic safety update reports (PSURs). These regular intervals are decided based on the risk profile of the products. Through these reports, the regulatory body collects all the data associated with the events and safety, primarily to provide a risk-benefit analysis of the product, and based on that, the mitigating actions are proposed (European Medicines Agency, 2013).

### 3.5 Surveillance methodologies

**FIGURE 4:**
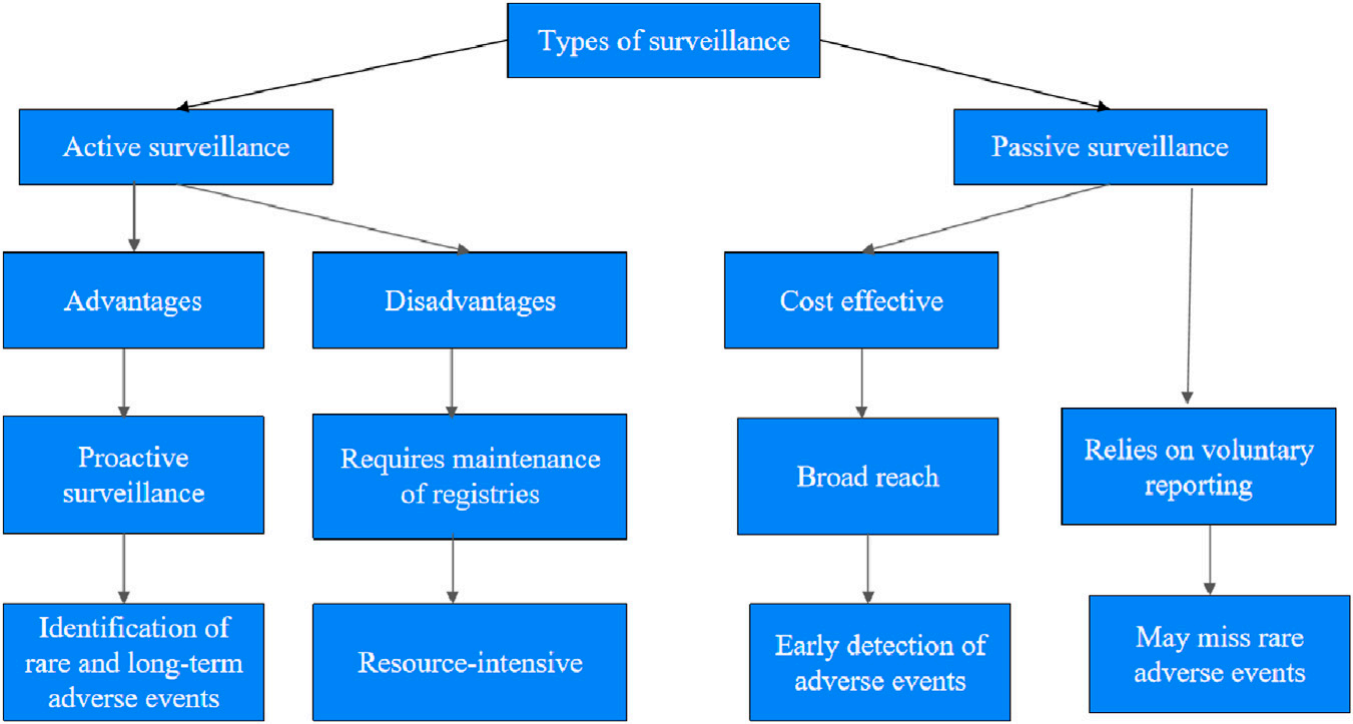
Advantages and Disadvantages of the two types of surveillance.

To conduct these extensive surveillance events, E.U.’s EMA broadly follows two methodologies (Figure 4).

#### 3.5.1 Active surveillance

Proactive surveillance and systematic collection of the data obtained through specific studies and monitoring programs from active surveillance. Under active surveillance, cohort studies are the primary source of the data generated by following the patients for any incidences or suspected adverse events. Active surveillance further requires the maintenance of registries or databases with the meticulous entries of the patients with their specific treatments to ensure long-term monitoring of the safety data. Through such design, active surveillance can identify and keep a record of rare and long-term adverse events.

#### 3.5.2 Passive surveillance

In addition to active surveillance, EU EMA also relies on the spontaneous collection and reporting of adverse events voluntarily by healthcare personnel, patients, manufacturers, and marketing authorization holders. Passive surveillance is cost-effective and has a broad reach compared to active surveillance. Additionally, considering the reporting at the manufacturer level, this method can be helpful for the early detection of new or even rare adverse events (Association of Health Care Journalists, 2025).

## 4 Post-marketing safety surveillance in the U.S.

### 4.1 Pharmacovigilance systems

#### 4.1.1 FDA Adverse Event Reporting System (FAERS)

Similar to the EU EMA’s EudraVigilance, the U.S. FDA has a centralized database known as the FDA Adverse Event Reporting System (FAERS). This database functions to collect and analyze reports of adverse events and medication errors related to drugs and therapeutic amenities. The sources for the data collection are mainly the adverse event reports from healthcare professionals, manufacturers, and patients or consumers. Based on this data, safety signals are decided by utilizing data mining and other advanced analytical tools. The risks that require investigations based on the signals obtained from the analyses are subjected to the risk management planning bodies. Additionally, the data is often shared with public repositories, such as the FDA website, allowing researchers, policymakers, and other stakeholders to analyse and offer management and solutions (FDA, 2023).

MAUDE, the FDA’s centralized database for medical devices, is designed to collect and monitor adverse events, malfunctions, and safety issues associated with medical devices, including the device components of drug-device combination products. It complements the FDA Adverse Event Reporting System (FAERS), which focuses on adverse drug reactions (ADRs) and safety signals related to the medicinal product component. Together, MAUDE and FAERS provide a comprehensive view of the safety profile of drug-device combination products. Unlike the EU system, the US system benefits from greater integration, as both MAUDE and FAERS are coordinated under the FDA’s oversight. This centralized approach reduces redundancies and allows for streamlined reporting and analysis of safety data, enhancing the ability to identify and address potential risks effectively.

#### 4.1.2 Role of the FDA’s Office of Surveillance and Epidemiology

Within the FDA, the lead center for drug review and evaluation called the Center for Drug Evaluation and Research (CDER), is majorly responsible for overseeing the medicinal therapeutic part of the combination product. Under CDER, the Office of Surveillance and Epidemiology (OSE) is specifically responsible for the monitoring work that is associated with the safety of marketed drugs or therapeutic biologics. The OSE fundamentally evaluates the safety data from the FAERS with the aim of identifying the potential risks associated with medicinal use. The evaluation and analysis are reported in the form of signals that are utilized for marketing prioritization of the products and subsequently communicated with the approval authorities. Those products that receive wrong signals are considered for regulatory actions that majorly include label changes, warnings, and, in worse cases, recall from the markets dependingon the safety evaluation. Even if the products make it to the market, post-marketing assessment studies and monitoring programs from the OSE further oversee and assess drug safety and long-term compliance. In the case of the combination product, OSE works with other lead centers of the FDA, like CDRH and CBER, to ensure the compliance of the medical devices with the FDA commitments (FDA, 2024).

### 4.2 Adverse event reporting requirements

Much like its E.U. counterpart, EudraVigilance, the FDA’s adverse event reporting is essential and a significant source of passive data collected by the OSE. There are three major requirements that OSE guidelines ensure compliance with when reporting adverse events. First is the duration of reporting the adverse events; for example, the severe and unexpected are required to be reported within 15 days of awareness, whereas others may be reported quarterly or annually. The second one is of paramount significance as it involves the format of the reporting to be detailed yet precise to provide a comprehensive report on the event, the product involved, and the patient outcomes observed. Finally, the reports are expected to be submitted electronically through the dedicated FAERS system for further analysis and record-keeping (FDA, 2023).

### 4.3 Risk evaluation and mitigation strategies (REMS)

Once the surveillance assessment report of a product is completed, it is approved only after ensuring that its benefits outweigh their associated risks. In case of safety signals, depending upon the severity of risks, the products may be recalled from the market. However, if there is scope for management of the risks, they are evaluated with recommendations for possible mitigation strategies. Risk evaluation and mitigation strategies (REMS) form a backbone for a majority of the under-achieving market products to become better and function in compliance with the promised purpose. There are various ways through which REMS can ensure the benefits outweigh the risks associated with a product. For example, medication guides must be up-to-date and detailed enough to inform patients about their safe use. Similarly, REMS may require the manufacturer to develop and implement communication plans to inform and guide healthcare providers regarding the known and reported risks associated with the products they recommend to patients. In addition to training, such as prescriber or healthcare setting certification programs, they may also require patient monitoring or restricted distribution programs; collectively these requirements are known as Elements to Assure Safe Use (ETASU). In summary, REMS offers a tailored guide to the specific risks associated with a medicinal product that is periodically monitored and even modified post-evaluation if necessary (FDA, 2025).

### 4.4 Periodic safety reports

There are fundamentally two types of periodic safety reports that are used to assess, analyse and decide the risk-benefit balance of a product: periodic adverse drug experience reports (PADERs) and periodic benefit-risk evaluation reports (PBRERs). PADERS primarily focus on the submission of the experiences associated with adverse drug events (Rossello, 2023) and are submitted quarterly for the first years of product evaluation, and annually following. PBRERs are comprehensive reports that aim to update the safety and risk-benefit balance and are submitted annually (Freyr, 2025).

### 4.5 Surveillance methodologies

The surveillance methodology of the FDA is more or less like the E.U.’s where the cohort-based studies, registries with detailed monitoring reports of the drug-specified patient, and utilization of electronic healthcare databases for product safety monitoring remain as the ground workers for the active surveillance. Active surveillance, through its robust and detailed safety data, helps to identify the rare and long-term adversities associated with product usage. Meanwhile, passive surveillance remains the cost-effective method to identify and suspect new or rare adverse events. Here, the spontaneous reporting voluntarily submitted by the healthcare professionals, manufacturers, and patients is also shared with FAERS, which further analyses them for their safety signals (FDA, 2023).

## 5 Key discrepancies in post-marketing safety surveillance

To summarize, although both the E.U.’s EMA and the U. S’s FDA function to ensure the safety and efficacy of the medicinal product, there are methodological differences in their regulatory frameworks for drug-device combination products. With respect to pharmacovigilance, both EudraVigilance, and FAERS provide a centralized database for the adverse event reports, but each system differs in scope and implementation. In the EU, EUDAMED complements EudraVigilance by centralizing safety data for the device components of combination products. Similarly, in the U.S., MAUDE handles device-related incidents, functioning alongside FAERS to provide a complete safety profile for combination products.

In the EU, multiple agencies are involved; the EMA works together with NCAs to regulate and conduct scientific assessments with proper risk management procedures in ensuring that the device components comply with the MDR. In the U.S., regulatory oversight is centralized under the FDA’s OCP, which coordinates between FAERS and MAUDE for drug- and device-related safety data. This streamlined approach reduces redundancies and improves regulatory efficiency. The OSE within the CDER evaluates the compliance and post-marketing studies.

When it comes to reporting the ADR reporting, both FAERS and EudraVigilance mandate reporting of severe events within 15 days, but timelines for non-serious events differ as follows: 90 days for EudraVigilance *versus* quarterly or even annually in some cases for FAERS. Device-related issues, such as malfunctions or field safety corrective actions (FSCAs), are reported to EUDAMED which is in implementation in the EU and MAUDE in the U.S. This provides additional insights into the safety of combination products. The more integrated system in the United States (US), where the FDA coordinates oversight through FAERS and MAUDE avoids the fragmentation and potential redundancies of the dual EudraVigilance and EUDAMED systems in the EU.

Furthermore, E.U.’s RMPs require manufacturers of all medicinal device products to outline their risk management strategies across the lifecycle of the product. In the U.S.’s REMS ensures the balance of risk benefits of the product, which is implemented through tailored measures such as prescriber training, patient monitoring, and restricted distribution programs like ETASU. Both systems require regular safety reports (PSURs in the EU, PADERs/PBRERs in the U.S.) to provide updates on risk-benefit assessments and ensure ongoing safety monitoring.

The differences in two regions’ perspectives are compared in Table 3.

**TABLE 3:** Comparison of regulatory frameworks and post-marketing safety surveillance for drug-device combination products in the EU and US.

Parameter	EU perspective	US perspective
Pharmacovigilance systems	Eudravigilance: Centralized database for adverse event reports managed by EMA. Sources include healthcare professionals, manufacturers, and patients. Data used to identify safety signals and manage risks. EUDAMED complements this by handling device-related incidents for combination products	FDA Adverse Event Reporting System (FAERS): Centralized database for adverse events and medication errors. Data from healthcare professionals, manufacturers, and patients. Safety signals identified using data mining and advanced analytical tools. Data shared with public repositories like the FDA website. MAUDE complements this by focusing on device-related issues. Both provide safety signals through data mining
Role of regulatory bodies	EMA and NCAs: EMA oversees medicinal products, collaborating with National Competent Authorities (NCAs) for evaluation and risk management	Office of Surveillance and Epidemiology (OSE): Within the Center for Drug Evaluation and Research (CDER) at the FDA, responsible for monitoring the safety of marketed drugs or therapeutic biologics. Evaluates safety data from FAERS and identifies potential risks, with regulatory actions including label changes, warnings, or recalls
Adverse event reporting requirements	Eudravigilance reporting: Severe events reported within 15 days; non-serious events within 90 days. Device-related issues reported to EUDAMED for further action	FAERS reporting: Severe events reported within 15 days; others quarterly or annually. Detailed and precise reporting required, submitted electronically through FAERS. Device malfunctions and incidents reported to MAUDE.
Risk evaluation and mitigation strategies (REMS)	Risk management plans (RMPs): Required for all medicinal products to outline lifecycle risk management strategies	REMS: Ensures benefits outweigh risks, including medication guides, communication plans with healthcare providers, prescriber training certification, patient monitoring, and restricted distribution programs (Elements to Assure Safe Use - ETASU). Tailored to specific risks, periodically monitored, and modified if necessary
Periodic safety reports	Periodic safety update reports (PSURs): Submitted regularly to update on safety and risk-benefit balance	Periodic adverse drug experience reports (PADERs): Focus on adverse drug experiences, submitted quarterly initially, then annually. Periodic benefit-risk evaluation reports (PBRERs): Comprehensive annual reports on the safety and risk-benefit balance
Surveillance methodologies	Active surveillance: Cohort- based studies, registries, and electronic healthcare databases. Identifies rare and long-term adverse effects. Passive Surveillance: Cost-effective method using spontaneous reporting from healthcare professionals, manufacturers, and patients	Active surveillance: Similar to EU, includes cohort-based studies, registries, and electronic healthcare databases. Identifies rare and long- term adverse effects. Passive Surveillance: Cost-effective method using spontaneous reporting from healthcare professionals, manufacturers, and patients, analyzed by FAERS for safety signals
Key discrepancies in post-marketing surveillance	Fragmented oversight: EMA, NCAs, and Notified Bodies share responsibilities, collaborative regulation and scientific assessment ADR Reporting: EudraVigilance: Centralised adverse event reporting for medicinal component. Dual systems (EudraVigilance and EUDAMED) operate independently, creating redundancies RMPs: Required for all products Strict Reporting: Severe events within 15 days. Non-serious events reported within 90 days	Centralized oversight: FDA and provides centralized oversight within CDER. Centralized ADR reporting: FDA coordinates pharmacovigilance via FAERS and device surveillance via MAUDE. Integration reduces redundancies and improves efficiency REMS: Ensures risk-benefit balance through ETASU components Reporting Flexibility: Severe events within 15 days; others quarterly or annually. Non-serious quarterly/annually

## 6 Challenges and implications

Although all the combination product regulatory bodies across the globe aim to ensure the efficacy and safety of the product, their discrepancies pose a significant challenge to their global marketing. This difference in the regulatory requirements has a direct influence on the complexity and the cost for the manufacturers, which is ultimately a burden on global healthcare. The primary reasons for the differences are regulatory divergencies that are rooted in the variation in definition, classification, and surveillance tactics, as we have observed in the case of EMA and FDA (Moseley et al., 2020). The complications are further elevated when there are formatting differences in the safety data submission and analysis, as EudraVigilance requires different formatting compared to FAERS. Furthermore, the phased implementation of EUDAMED limits public access to comprehensive safety data for combination products. The integrated FAERS and MAUDE provide not only a streamlined approach but is also integrated with broad public access to safety data, enabling researchers, healthcare professionals, and policymakers to analyze trends and develop risk mitigation strategies.

The distinct approaches in the risk management and signal identification criteria followed by RMPs and REMS become a hurdle. Due to these factors suggestive of a lack of harmonization, manufacturers often are subjected to compliance costs that involve following different regulatory landscapes, duplicate costs and resource allocations for the approval of the same product. This is usually the main reason for the market delays of the products that, even after launching, are subjected to numerous regulatory uncertainties. Such factors also severely hamper global innovation and have been recognized as one of the significant causes of technology transfer in the objectives of international access. Apart from the derogatory impact of the lack of synergistic regulation on the manufacturers or industries, patients are ultimately the victims. Inconsistencies in the regulatory bodies result in inefficient monitoring and, thereby, identification of the safety issues that could directly affect the patient’s safety.

Furthermore, the variations in the global safety signals might mislead the signals due to the lack of joint adverse event detecting agencies (Raj et al., 2019). This will ultimately lead to mistrust of the patient in the innovation and hard-earned product by the manufacturers. Keeping these differences in view, future directions must be invested in the convergence of the regulatory aspects associated with the combination products. With at least three primary goals in mind, 1) global regulatory bodies must develop standard guidelines that are valid globally, 2) transparency in terms of data sharing must be implicated for worldwide awareness and 3) potentiating cumulative solutions that must result in the common harmonized databases related to the usage of combination products. These goals can be achieved gradually through international conferences aiming at awareness and the need for common regulation for global advancement in healthcare. Bilateral agreements with mutual recognition must be established between regulatory bodies and global stakeholders to understand the challenges and work on feasible solutions.

## 7 Conclusion

Although both the U.S. and the E.U. employ a robust regulatory framework, from capable pharmacovigilance systems to risk management strategies, they differ significantly in their methodology. The dual reporting requirement in the European Union (EU), where drug-related and device-related incidents are reported to EudraVigilance and EUDAMED, respectively, creates fragmentation and redundancies compared to the more integrated system in the United States (US), where the FDA coordinates oversight through FAERS and MAUDE. This difference has been recognized globally as a significant challenge for harmonization that severely impacts innovation, manufacturing, and, ultimately, patient safety. Addressing the regulatory discrepancies is crucial to ensure the safety of the product and its global access. Such an improvement is possible by understanding the merits of the unification of the regulatory bodies achievable by enhanced communication, standard repositories and agreements with mutual recognition. It is only through continued efforts towards collaboration, standardization, and innovation that the global regulatory bodies can ensure the cumulative safety and well-being of patients worldwide.
